# Risk factors for aspiration pneumonia during concurrent chemoradiotherapy or bio-radiotherapy for head and neck cancer

**DOI:** 10.1186/s12885-020-6682-1

**Published:** 2020-03-04

**Authors:** Hiromichi Shirasu, Tomoya Yokota, Satoshi Hamauchi, Yusuke Onozawa, Hirofumi Ogawa, Tsuyoshi Onoe, Tetsuro Onitsuka, Takashi Yurikusa, Keita Mori, Hirofumi Yasui

**Affiliations:** 1 Shizuoka Cancer Center, Division of Gastrointestinal Oncology, 1007 Shimonagakubo Nagaizumi-cho Sunto-gun, Shizuoka, 411-8777 Japan; 2 Shizuoka Cancer Center, Division of Medical Oncology, Sunto-gun, Shizuoka, Japan; 3 Shizuoka Cancer Center, Division of Radiation Oncology and Proton Therapy, Sunto-gun, Shizuoka, Japan; 4 Shizuoka Cancer Center, Division of Head and Neck Surgery, Sunto-gun, Shizuoka, Japan; 5 Shizuoka Cancer Center, Division of Dentistry and Oral Surgery, Sunto-gun, Shizuoka, Japan; 6 Shizuoka Cancer Center, Clinical Research Center, Sunto-gun, Shizuoka, Japan

**Keywords:** Head and neck cancer, Aspiration pneumonia, Chemoradiotherapy, Radiotherapy, Risk factors

## Abstract

**Background:**

Aspiration pneumonia is one of the most important side effects of chemoradiotherapy (CRT) and bio-radiotherapy (BRT) in patients with head and neck cancer (HNC). Aspiration pneumonia can lead to cancer-related mortality in HNC patients. However, the relationship between aspiration pneumonia occurring during CRT or BRT for HNC and treatment outcomes in HNC patients is not well characterized. In this study, we assessed the influence of aspiration pneumonia on treatment outcomes and sought to identify the clinical risk factors for aspiration pneumonia during definitive CRT and BRT in HNC patients.

**Methods:**

We retrospectively assessed the data pertaining to patients with locally advanced HNC who received definitive CRT or BRT at the Shizuoka Cancer Center between August 2006 and December 2016.

**Results:**

Among the 374 HNC patients who received CRT or BRT, 95 (25.4%) developed aspiration pneumonia during treatment. Aspiration pneumonia was significantly associated with therapeutic response to CRT or BRT (multivariate adjusted odds ratio for complete response, 0.52, *p* = 0.020) and poor overall survival (multivariate adjusted hazard ratio for overall survival, 1.58, *p* = 0.024). The multivariate analyses identified four independent factors for aspiration pneumonia: poor oral hygiene, high N-classification, hypoalbuminemia before treatment, and inpatient treatment.

**Conclusions:**

Aspiration pneumonia occurring during CRT or BRT has a detrimental effect on the therapeutic response and survival of HNC patients. Careful attention should be paid to these risk factors for aspiration pneumonia in HNC patients undergoing CRT or BRT.

## Background

Radiotherapy (RT) plays a central role in the treatment of head and neck cancers (HNCs). Definitive chemoradiotherapy (CRT) with curative intent is a common approach to treat locally advanced HNC with the goal of organ preservation [1, 2]. Bio-radiotherapy (BRT), which is RT administered in combination with cetuximab, is regarded as a treatment option for patients with locally advanced HNCs [3]. CRT and BRT are superior to radical surgery with respect to maintenance of organ function and the quality of life of the patient. However, CRT and BRT are invariably associated with adverse effects such as aspiration pneumonia, mucositis, xerostomia, dysphagia, and hematological toxicity. These side effects may necessitate unplanned breaks and delay in RT administration, leading to poorer outcomes [4–8]. Therefore, appropriate management against acute toxicities is required for patients cured by CRT. In particular, aspiration pneumonia refers to the pulmonary consequences that result from the abnormal entry of fluid, particulate exogenous substances, or endogenous secretions into the lower airways [9]. In a prospective study, aspiration pneumonia occurred in up to 62% of patients one year after therapy [10]; several retrospective studies have reported an incidence of approximately 25% after CRT or BRT [11, 12]. Studies have indicated that aspiration pneumonia is a major cause of post-treatment morbidity and death in HNC patients [13]. Although a few studies have investigated aspiration pneumonia during treatment [14], the incidence or risk factors of aspiration pneumonia in patients receiving CRT and BRT are not well characterized. Therefore, the aim of this study was to assess the effect of aspiration pneumonia during definitive CRT and BRT on treatment outcomes and to identify the clinical risk factors for aspiration pneumonia in HNC patients.

## Methods

### Patients

This study used medical records to identify 374 patients with locally advanced HNC who received definitive concurrent CRT or BRT at the Shizuoka Cancer Center between August 2006 and December 2016. Patients who had recurrent or metastatic lesions or those who received resection of the primary tumor before CRT were excluded. Patients who had other coexisting primary cancers in addition to HNC were included only if the HNC was deemed to have had the most significant impact on their prognosis. Shizuoka Cancer Center Institutional Review Board approved this study; informed consent was obtained from all patients.

### Study variables

We retrospectively reviewed the data pertaining to the incidence of aspiration pneumonia, the time of onset of aspiration pneumonia, and treatment efficacy. The background variables for risk factors for aspiration pneumonia included age, gender, Eastern Cooperative Oncology Group (ECOG) performance status, primary tumor site, body mass index (BMI), TNM staging defined by the American joint Committee on Cancer/Union for International Cancer control staging classification (7th edition), tumor histology, the Brinkman index (defined as the number of cigarettes smoked per day times the number of smoking years), habitual alcoholic consumption, consumption of proton pump inhibitors or histamine H2-receptor antagonist (H2 blockers), consumption of angiotensin II receptor blockers or angiotensin-converting enzyme inhibitors, consumption of sleeping pills, oral hygiene, coexistence of other malignancies before treatment, the Charlson comorbidity index, and serum albumin (ALB) and hemoglobin (Hb) values before treatment. Habitual alcoholic consumption was defined as drinking more than four days a week. Poor oral hygiene was defined as the presence of middle level or more dental plaques as diagnosed by a dentist or a dental hygienist. The Charlson comorbidity index is used to predict morbidity and mortality in several clinical conditions. This index consists of three parts: disease assessment including 16 diseases including neurological disorders, severity assessment, and scoring [15].. We also reviewed the following treatment-related variables: the presence or absence of induction chemotherapy, percutaneous endoscopic gastrostomy prior to treatment, inpatient or outpatient treatment, chemotherapy regimen, radiation technique (conventional three-dimensional conformal radiation therapy [3D-CRT] or intensity-modulated radiation therapy [IMRT]), irradiation field (local or whole neck), radiation dose, treatment efficacy evaluated according to the Response Evaluation Criteria in Solid Tumors (RECIST) version 1.1 [complete response (CR) or non-CR] [16], mucositis during treatment evaluated by the Common Terminology Criteria for Adverse Events version 4.0, and dysphagia score during treatment [17].

### Definition of aspiration pneumonia

In this study, symptomatic aspiration pneumonia was defined as a clinical condition meeting all of the following criteria as mentioned before in our previous study on aspiration pneumonia after CRT or BRT [12]: (1) patients had both subjective and objective symptoms suggesting pneumonia. The subjective symptoms included wet cough, sputum, and fever. The objective symptoms included the presence of coarse crackles in the chest, elevated levels of inflammatory markers (e.g., white blood cell count or C-reactive protein), or imaging findings (e.g., infiltration on chest X-ray or consolidation in chest computed tomography). (2) The presence of aspiration pneumonia was suspected clinically (choking or delayed swallowing) or by endoscopic or video-fluorographic examination. (3) Bacterial culture or urine antigen tests showing no evidence of microorganisms that cause atypical pneumonia, such as Legionella or Mycoplasma.

### Statistical analysis

The cumulative incidence of aspiration pneumonia was measured using the Kaplan-Meier method. The time to event was measured from the date of the first RT to the date of the event. The association between clinical covariates and the incidence of aspiration pneumonia or treatment efficacy was assessed by univariate analysis using Fisher’s exact test; variables that showed a significant association on univariate analysis were further analyzed using a multivariate logistic regression model.

The overall survival (OS) time was measured from the date of the first RT to the date of death from any cause or to the last date of confirmed survival. Survival curves were generated using the Kaplan-Meier method. Log-rank test was used to evaluate between-group with respect to survival. Variables that showed a significant association with survival on univariate analysis were included in the multivariate analysis using the Cox regression model.

All statistical tests were two-sided and *p*-values < 0.05 were considered indicative of statistical significance. All statistical analyses were conducted using the EZR version 1.32 (Saitama Medical Center, Jichi Medical University, Saitama Japan) [18].

## Results

### Patient selection and characteristics

The patients’ characteristics and delivery of treatment are presented in Table 1: 91 (24%) patients had a primary site classified as N2c or worse. Oral hygiene before treatment was poor in 183 (52%) patients. Serum albumin levels before treatment were below the normal range in 61 (16%) patients. A total of 189 patients received outpatient treatment. Additionally, 45 patients had coexisting malignancies such as multiple primary HNC and esophageal, gastric, renal, prostate, or lung cancer. All of these cancers were detected at an early stage by routine endoscopic or computed tomography screening.

**Table 1:** Patients’ characteristics

Background	n (%)
Age	
< 70 years	275 (74)
≥ 70 years	99 (26)
Gender	
Male	322 (86)
Female	52 (14)
ECOG performance status	
0	234 (63)
1	121 (32)
2	16 (4)
3	3 (1)
Body mass index	
< 20	97 (26)
≥ 20	277 (74)
Primary site	
Larynx	57 (15)
Nasopharynx	48 (13)
Hypopharynx	132 (34)
Nasal sinus	21 (6)
Oropharynx	101 (27)
Oral cavity	14 (4)
Ear canal	1 (1)
T-classification	
1	32 (9)
2	136 (36)
3	86 (23)
4a	92 (25)
4b	28 (7)
N-classification	
0	76 (20)
1	54 (15)
2a	19 (5)
2b	134 (36)
2c	75 (20)
3	16 (4)
Tumor histology	
SCC	347 (93)
Others	27 (7)
Brinkman index	
< 500	131 (35)
≥ 500	243 (65)
Habitual alcoholic consumption	
Yes	221 (59)
No	153 (41)
Use of ACEi or ARB	
Yes	69 (18)
No	305 (82)
Use of PPI or H2 blocker	
Yes	198 (53)
No	176 (47)
Oral hygiene before treatment	
Good	179 (48)
Poor	183 (52)
Coexistence of other malignancies	
Yes	45 (12)
No	329 (88)
Charlson comorbidity index	
0–1	293 (78)
≥ 2	81 (22)
Serum albumin before treatment	
Within normal limits	313 (84)
Less than normal range	61 (16)
Hemoglobin before treatment	
Within normal limits	265 (71)
Less than normal range	109 (29)
Use of sleeping pills before treatment?	
Yes	185 (49)
No	189 (51)
Induction chemotherapy	
Yes	97 (26)
No	277 (74)
Concurrent chemotherapy regimen	
CDDP-based	278 (74)
CBDCA-based	64 (17)
Cetuximab	32 (9)
Radiation technique	
Conventional 3D-CRT	253 (68)
IMRT	121 (32)
Radiation dose, Gy	
70Gy	363 (97)
60-70Gy	3 (1)
< 60Gy	8 (2)
Irradiation field	
Local	67 (18)
Whole neck	307 (82)
Percutaneous endoscopic gastrostomy prior to treatment	
Yes	229 (61)
No	155 (39)
Treatment	
Inpatient	185 (49)
Outpatient	189 (51)

### The frequency and time to onset of aspiration pneumonia

**Fig. 1:**
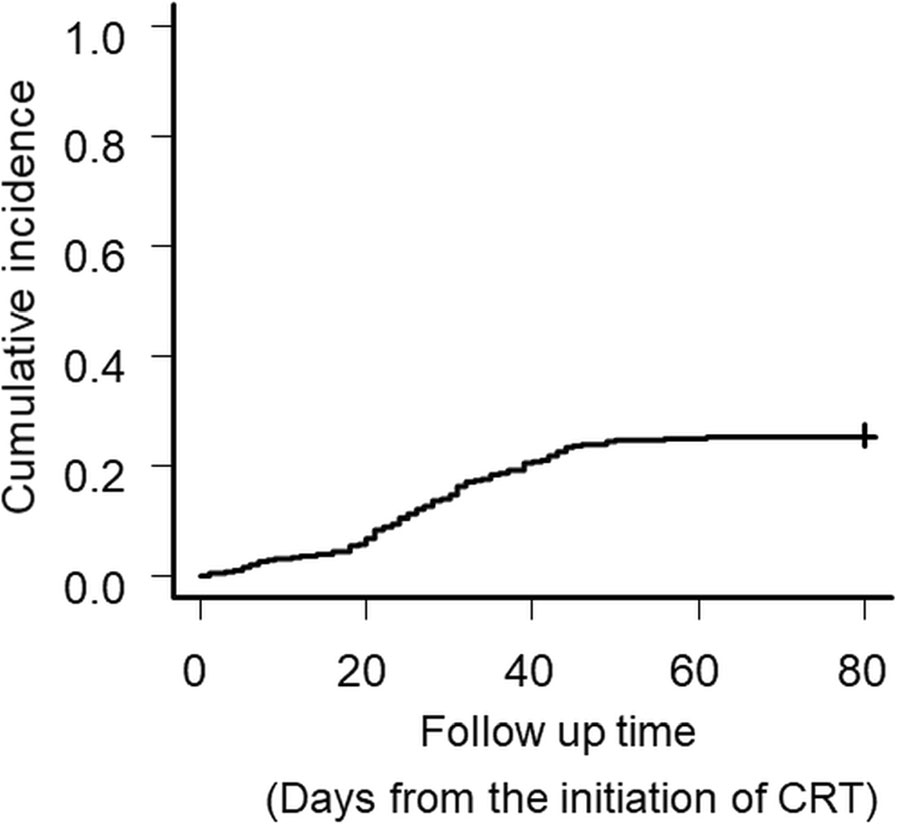
Cumulative incidence of aspiration pneumonia

Among the 374 patients with locally advanced HNC, 95 (25.4%) developed aspiration pneumonia during CRT or BRT. Figure 1 displays a Kaplan-Meier curve exhibiting the cumulative risk of aspiration pneumonia. The median time from the date of the first RT to the date of developing aspiration pneumonia was 28 days (range 1–61).

### Treatment compliance of CRT or BRT

Among the 95 patients with aspiration pneumonia, treatment interruptions or unplanned breaks during CRT or BRT occurred in 34 patients (36%). In contrast, among the 279 patients who did not develop aspiration pneumonia, only 8 patients (3%) experienced treatment interruption or unplanned breaks during CRT or BRT. Thus, treatment interruption or unplanned breaks were significantly more frequent in patients with aspiration pneumonia than those without aspiration pneumonia (*p* < 0.01).

### Risk factors for aspiration pneumonia

Univariate and multivariate analyses identified four independent risk factors for aspiration pneumonia: advanced N-classification (2c-3) [multivariate adjusted odds ratio (OR) 1.96, 95% confidential interval (CI) 1.08–3.57, *p* = 0.027], poor oral hygiene (OR 2.08, 95% CI 1.20–3.57, *p* = 0.0076), hypoalbuminemia before treatment (OR 2.78, 95% CI 1.37–5.56, *p* = 0.0015), and inpatient treatment (OR 2.35, 95% CI 1.39–3.98, p = 0.0015) (Table 2).

**Table 2:** Univariate and multivariate analysis for risk factors of aspiration pneumonia

Variables	Univariate analysis			Multivariate analysis		
	Odds ratio	95% CI	P	Odds ratio	95% CI	P
Age < 70 vs. ≥70	0.94	0.56–1.59	0.82			
Gender Male vs. Female	1.32	0.65–2.68	0.45			
ECOG Performance status 0–1 vs.2–3	0.28	0.11–0.72	**0.0081**	0.68	0.24–1.93	0.47
BMI < 20 vs. ≥20	1.56	0.94–2.60	0.086			
Primary site Oropharynx vs. others	1.77	1.07–2.92	**0.027**	0.84	0.47–1.49	0.55
T-classification 1–2 vs. 3–4	0.86	0.54–1.37	0.52			
N-classification 0-2b vs. 2c-3	0.39	0.23–0.66	**< 0.001**	0.51	0.28–0.93	**0.027**
Histology SCC vs. others	1.54	0.57–4.18	0.40			
Brinkman index < 500 vs. ≥500	1.11	0.69–1.81	0.67			
Habitual alcoholic consumption Yes vs. No	1.43	0.88–2.33	0.16			
Use of ACEi or ARB Yes vs. No	1.25	0.70–2.22	0.45			
Use of PPI or H2 blocker Yes vs. No	1.39	0.86–2.22	0.18			
Oral hygiene before treatment Good vs. Poor	0.40	0.25–0.66	**< 0.001**	0.48	0.28–0.83	**0.0076**
Coexistence of other malignancies Yes vs. No	1.12	0.78–1.56	0.57			
Charlson comorbidity index 0–1 vs. ≥2	0.76	0.44–1.31	0.32			
Serum albumin before treatment Within normal limits vs. less than normal range	0.15	0.27–0.48	**< 0.001**	0.36	0.18–0.73	**0.0015**
Hemoglobin before treatment Within normal limits vs. less than normal range	0.37	0.23–0.61	**< 0.001**	0.78	0.43–1.43	0.42
Use of sleeping pills before treatment Yes .vs No	1.35	0.75–2.44	0.32			
Induction chemotherapy Yes vs. No	0.76	0.44–1.31	0.33			
Concurrent chemotherapy regimen CDDP vs. others	1.30	0.75–2.25	0.36			
Radiation technique Conventional 3D-CRT vs. IMRT	1.12	0.68–1.85	0.66			
Radiation dose 70Gy vs. <70Gy	0.58	0.17–2.04	0.40			
Irradiation field Local vs. whole neck	0.40	0.19–0.84	**0.016**	0.56	0.25–1.23	0.15
Percutaneous endoscopic gastrostomy prior to treatment Yes vs. No	1.62	0.96–2.77	0.067			
Treatment Inpatient vs. Outpatient	2.70	1.65–4.40	**< 0.001**	2.35	1.39–3.98	**0.0015**
Dysphagia score before treatment 1–2 vs. 3–4	0.56	0.30–1.03	0.062			

### Correlation between treatment efficacy and aspiration pneumonia

**Fig. 2:**
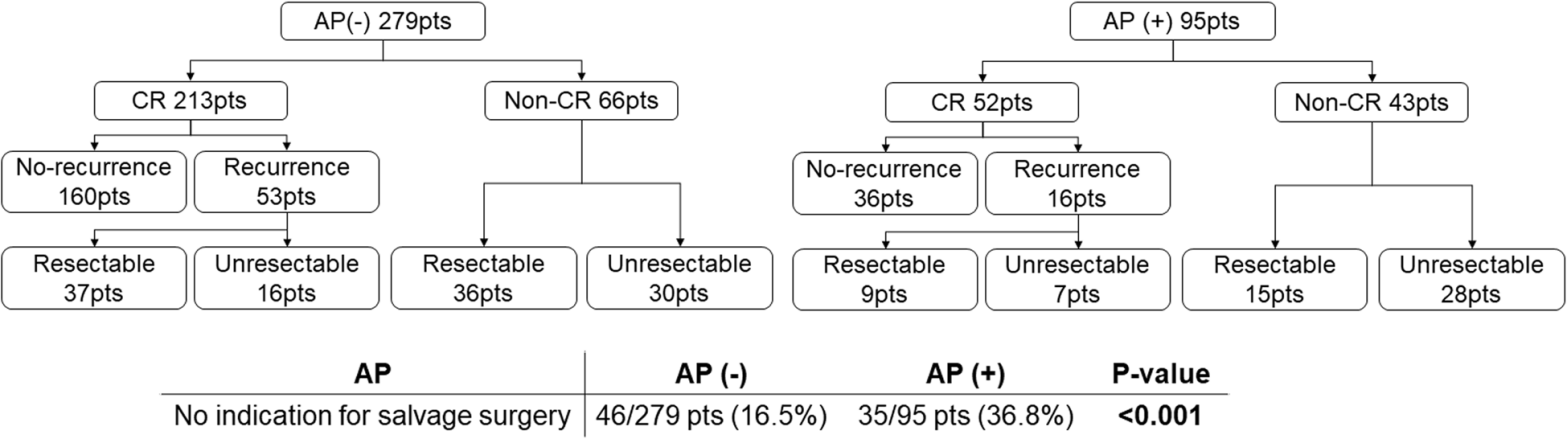
The treatment flow diagram according to the occurence of aspiration pneumonia. AP: aspiration pneumonia, CR: complete response, pts: patients

Next, we investigated the correlation between treatment efficacy and the occurrence of aspiration pneumonia. Univariate and multivariate analyses identified aspiration pneumonia as independent predictive factor for CR (multivariate adjusted OR 0.52, 95% CI 0.33–0.90, *p* = 0.020) (Table 3). CR induced by CRT or BRT was observed in 71% patients (265/374). The CR rate among patients without aspiration pneumonia (76%; 213/279) was significantly greater than that among patients with aspiration pneumonia (55%; 52/95). The treatment flow diagram according to the presence or absence of aspiration pneumonia is summarized in Fig. 2. Among the 213 patients without aspiration pneumonia who achieved CR, 53 experienced recurrence and 16 underwent non-R0 salvage surgery. Among the 66 patients who did not achieve CR, 30 underwent non-R0 salvage surgery. Among the 52 patients with aspiration pneumonia who achieved CR, 16 experienced recurrence and seven received non-R0 salvage surgery. Among the 43 patients who did not achieve CR, 28 underwent non-R0 salvage surgery. Thus, the frequency of patients who did not require R0 salvage surgery was significantly lower in the group without aspiration pneumonia than in the group with aspiration pneumonia [16.5% (46/279) vs. 36.8% (35/95), *p* < 0.001].

**Table 3:** Univariate and multivariate analysis for factors of complete response

Variables	Univariate analysis			Multivariate analysis		
	Odds ratio	95% CI	P	Odds ratio	95% CI	P
Age < 70 vs. ≥70	1.23	0.76–2.01	0.40			
Gender Male vs. Female	0.80	1.41–1.53	0.49			
ECOG Performance status 0–1 vs.2–3	4.14	1.58–10.8	**0.0037**	2.30	0.81–6.52	0.12
BMI < 20 vs. ≥20	0.51	0.62–7.47	0.227			
Primary site Oropharynx vs. others	1.02	0.62–1.68	0.94			
T-classification 1–2 vs. 3–4	1.97	1.25–3.12	**0.0035**	1.56	0.95–2.57	0.082
N-classification 0-2b vs. 2c-3	2.31	1.43–3.73	**< 0.001**	1.57	0.90–2.73	0.11
Histology SCC vs. others	0.36	0.12–1.08	0.069			
Brinkman index < 500 vs. ≥500	1.16	0.73–1.84	0.54			
Habitual alcoholic consumption Yes vs. No	0.64	0.40–1.01	0.055			
Use of ACEi or ARB Yes vs. No	0.97	0.55–1.69	0.91			
Use of PPI or H2 blocker Yes vs. No	1.14	0.73–1.75	0.59			
Oral hygiene before treatment Good vs. Poor	1.58	1.01–2.48	**0.043**	1.11	0.68–1.83	0.68
Coexistence of other malignancies Yes vs. No	0.89	0.64–1.23	0.48			
Charlson comorbidity index 0–1 vs. ≥2	1.32	0.79–2.22	0.29			
Serum albumin before treatment Within normal limits vs. less than normal range	2.78	1.59–4.77	**< 0.001**	1.88	0.95–3.75	0.069
Hemoglobin before treatment Within normal limits vs. less than normal range	1.75	1.09–2.79	**0.020**	1.07	0.60–1.91	0.82
Use of sleeping pills before treatment Yes .vs No	0.63	0.41–0.98	**0.042**	0.75	0.39–1.43	0.38
Induction chemotherapy Yes vs. No	1.15	0.69–1.89	0.60			
Concurrent chemotherapy regimen CDDP vs. others	1.15	0.70–1.89	0.57			
Radiation technique Conventional 3D-CRT vs. IMRT	0.60	0.37–0.98	**0.043**	0.65	0.38–1.12	0.12
Radiation dose 70Gy vs. <70Gy	4.13	1.18–14.4	**0.026**	3.70	0.97–14.1	0.056
Irradiation field Local vs. whole neck	0.98	0.56–1.72	0.95			
Percutaneous endoscopic gastrostomy prior to treatment Yes vs. No	0.50	0.31–0.80	**0.041**	0.68	0.40–1.15	0.15
Treatment Inpatient vs. Outpatient	0.62	0.40–0.96	**0.032**	0.86	0.52–1.40	0.53
The worst mucositis grade during treatment 1–2 vs. 3–4	1.09	0.70–1.70	0.70			
The worst dysphagia score during treatment 1–2 vs. 3–4	1.39	0.89–2.17	0.15			
Aspiration pneumonia during treatment Yes vs. No	0.38	0.23–0.62	**< 0.001**	0.52	0.30–0.90	**0.020**

### Correlation between survival and aspiration pneumonia

**Fig. 3:**
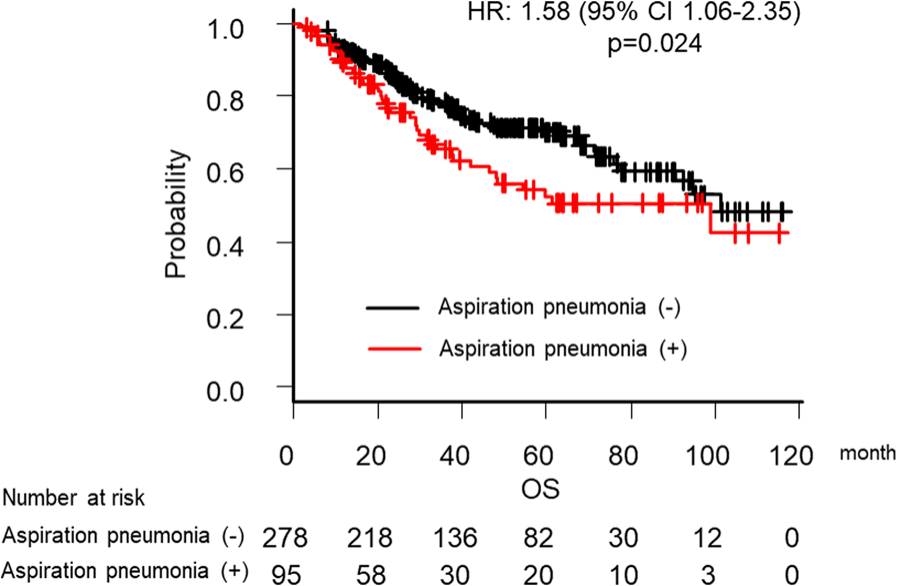
Adjusted Kaplan-Meier curve illustrating overall survival from the date of initiation of chemoradiotherapy or bio-radiotherapy stratified according to whether they developed aspiration pneumonia. Vertical dashes indicate censored observations. HR: hazard ratio, CI: confidence interval, OS: overall survival

We further investigated the correlation between OS and the occurrence of aspiration pneumonia (Table 4). Univariate and multivariate analyses revealed eight independent prognostic factors for OS: younger age [multivariate adjusted hazard ratio (HR) 0.64, 95% CI 0.43–0.95, *p* = 0.026], male gender (HR 2.47, 95% CI 1.27–4.81, *p* = 0.0080), low BMI (HR 1.53, 95% CI 1.03–2.28, *p* = 0.035), advanced T-classification (HR 1.72, 95% CI 1.15–2.63, *p* = 0.0087), advanced N-classification (HR 1.82, 95% CI 1.19–2.70, *p* = 0.0050), hypoalbuminemia before treatment (HR 2.00, 95% CI 1.20–3.33, *p* = 0.0069), low radiation dose (HR 5.56, 95% CI 2.50–11.9, *p* < 0.001), and aspiration pneumonia (HR 1.58, 95% CI 1.06–2.35, *p* = 0.024). Survival curves adjusted for the covariates from a Cox proportional hazard model indicated that the occurrence of aspiration pneumonia was significantly associated with the risk of death (Fig. 3).

**Table 4:** Univariate and multivariate analysis for overall survival

Variables	Univariate analysis			Multivariate analysis		
	Odds ratio	95% CI	P	Odds ratio	95% CI	P
Age < 70 vs. ≥70	0.61	0.42–0.88	**0.0083**	0.64	0.43–0.95	**0.026**
Gender Male vs. Female	1.99	1.07–3.69	**0.029**	2.47	1.27–4.81	**0.0080**
ECOG Performance status 0–1 vs.2–3	0.31	0.17–0.55	**< 0.001**	0.94	0.461.93	0.87
BMI < 20 vs. ≥20	1.72	1.20–2.50	**0.0043**	1.53	1.03–2.28	**0.035**
Primary site Oropharynx vs. others	1.25	0.85–1.83	0.25			
T-classification 1–2 vs. 3–4	0.42	0.28–0.61	**< 0.001**	0.58	0.38–0.87	**0.0087**
N-classification 0-2b vs. 2c-3	0.50	0.34–0.74	**< 0.001**	0.55	0.37–0.84	**0.0050**
Histology SCC vs. others	2.06	0.84–5.06	0.11			
Brinkman index < 500 vs. ≥500	0.95	0.66–1.36	0.76			
Habitual alcoholic consumption Yes vs. No	1.22	0.85–1.72	0.29			
Use of ACEi or ARB Yes vs. No	0.97	0.63–1.49	0.88			
Use of PPI or H2 blocker Yes vs. No	1.20	0.85–1.72	0.30			
Oral hygiene before treatment Good vs. Poor	0.66	0.46–0.94	**0.021**	0.94	0.65–1.37	0.76
Coexistence of other malignancies Yes vs. No	1.16	0.90–1.52	0.24			
Charlson comorbidity index 0–1 vs. ≥2	0.62	0.41–0.92	**0.018**	0.72	0.47–1.11	0.14
Serum albumin before treatment Within normal limits vs. less than normal range	0.33	0.22–0.48	**< 0.001**	0.50	0.30–0.83	**0.0069**
Hemoglobin before treatment Within normal limits vs. less than normal range	0.58	0.40–0.83	**0.0028**	0.83	0.53–1.31	0.43
Use of sleeping pills before treatment Yes .vs No	2.00	1.39–2.86	**< 0.001**	1.16	0.73–1.85	0.54
Induction chemotherapy Yes vs. No	0.87	0.56–1.33	0.51			
Concurrent chemotherapy regimen CDDP vs. others	0.66	0.45–0.97	**0.036**	0.71	0.45–1.12	0.14
Radiation technique Conventional 3D-CRT vs. IMRT	1.26	0.83–1.92	0.27			
Radiation dose 70Gy vs. <70Gy	0.25	0.12–0.50	**< 0.001**	0.18	0.084–0.40	**< 0.001**
Irradiation field Local vs. whole neck	0.90	0.56–1.43	0.66			
Percutaneous endoscopic gastrostomy prior to treatment Yes vs. No	1.76	1.21–2.56	**0.0034**	1.16	0.78–1.73	0.47
Treatment Inpatient vs. Outpatient	1.77	1.24–2.53	**0.0018**	1.47	0.99–2.17	0.052
The worst mucositis grade during treatment 1–2 vs. 3–4	0.98	0.68–1.40	0.90			
The worst dysphagia score during treatment 1–2 vs. 3–4	0.92	0.64–1.31	0.63			
Aspiration pneumonia during treatment Yes vs. No	1.56	2.22–3.23	**< 0.001**	1.58	1.06–2.35	**0.024**

## Discussion

The treatment goal of CRT or BRT for patients with locally advanced HNC is to cure the patient. However, aspiration pneumonia during CRT or BRT frequently necessitates treatment interruption or unplanned breaks in radiotherapy; this adversely affects the therapeutic outcomes including cure rates, durability of remission, and patient survival [19]. Therefore, development of strategies for prevention of aspiration pneumonia during CRT or BRT is a key imperative to maintain treatment compliance. The current study revealed a substantial incidence (25.4%) of aspiration pneumonia during CRT or BRT. We identified four independent risk factors for aspiration pneumonia: advanced N-classification (N2c-N3), poor oral hygiene, hypoalbuminemia before treatment, and inpatient treatment. Previous studies have identified several risk factors for aspiration pneumonia in patients with HNC after completing CRT [10–12, 20]; however, to the best of our knowledge, this study is the first to investigate the risk factors for aspiration pneumonia during CRT or BRT.

The reported incidence of aspiration pneumonia ranges from 5 to 23.8% [11, 12, 21]. The cumulative incidence of aspiration pneumonia in our study is somewhat higher than that in previous reports. This may be attributable to differences with respect to patient characteristics, duration of follow-up, and definition of aspiration pneumonia used in previous studies. For instance, in a study by Mortensen et al., approximately 5% HNC patients receiving radiotherapy alone developed pneumonia within 1 year after receiving radiotherapy [20]. On the other hand, 23.8% of patients with HNC developed aspiration pneumonia within 5 years after receiving radiotherapy with concurrent chemotherapy (CRT) [11]. Furthermore, in our previous study, 21.3% of patients with HNC developed aspiration pneumonia after CRT or radiotherapy with concurrent cetuximab (BRT) [12]. These findings suggest that the combination of chemotherapy or cetuximab with radiotherapy may be associated with a higher risk of aspiration pneumonia. Thus, the frequency of aspiration pneumoniae depends on the presence or absence of concurrent use of chemotherapy; the frequency observed in the current study is consistent with previous studies and may be acceptable in our clinical practice.

Advanced T and N stages are known to be associated with greater impairment of swallowing [22, 23]. Langius et al. reported that patients with advanced N-stage require irradiation of major salivary glands, which leads to xerostomia, acute dysphagia, and impaired swallowing [24]. These adverse effects may cause malnutrition and dehydration, which increases the risk of aspiration pneumonia [23]; this is because malnutrition reduces the resistance to infection by depressing the immune system, and dehydration decreases the salivary flow, which promotes altered colonization of the oropharynx [25].

Several studies have demonstrated that oral care is associated with a decreased incidence of aspiration pneumonia in elderly people [26–28]. We earlier reported that a systematic oral care program for patients with HNC may improve treatment compliance by decreasing the risk of infection [29]. Although we did not assess the efficacy of oral care in preventing aspiration pneumonia during CRT or BRT, the pre-assessment of oral hygiene by dentists and/or dental hygienists may play an important role in the prediction of aspiration pneumonia.

The interaction between nutritional status and the immune system has been emphasized. Poor nutrition increases the host susceptibility to infection and may trigger a vicious cycle leading to further aggravation of malnutrition [30]. Indeed, malnutrition (defined as a serum albumin level of < 2.5 g/dL) was identified as a predictor of aspiration pneumonia after RT alone [31]. Furthermore, our previous study identified hypoalbuminemia as a risk factor for aspiration pneumonia after CRT or BRT [12]. Consistent with these reports, the present study also identified hypoalbuminemia as a risk factor for aspiration pneumonia during CRT or BRT.

Our study observed an increased risk of aspiration pneumonia in patients who received CRT or BRT in the inpatient setting. Previous studies indicated an increased risk of aspiration pneumonia in patients who received treatment at teaching hospitals, which may reflect the differences with respect to unmeasured patient characteristics, such as more comorbidities and worse general condition [11]. To adjust for these factors, we conducted a multivariate analysis including the Charlson comorbidity index. However, the Charlson comorbidity index was not identified as a risk factor for aspiration pneumonia. Besides, there was no difference between inpatient treatment group and outpatient treatment group in terms of patients’ background such as performance status and Charlson comorbidity index. Therefore, the increased risk of aspiration pneumonia in patients who received treatment in the hospital may be attribute to the greater likelihood of detection of aspiration pneumonia owing to closer monitoring of patients in the inpatient setting.

Based on this analysis, systematic evaluation of the four risk factors before and during treatment may help prevent aspiration pneumonia in patients undergoing CRT or BRT [23]. Besides, multidisciplinary intervention by medical staff is indispensable to perform this program. For instance, dentists and dental hygienists should be involved in routine oral screening, oral care and continuous evaluation of oral hygiene. The speech and swallowing rehabilitation team should evaluate aspiration using video-fluoroscopic examination and institute rehabilitation measures. Appropriate evaluation and intervention by multidisciplinary team may help improve treatment outcomes [32].

Our study also demonstrated a strong correlation between the occurrence of aspiration pneumonia and treatment efficacy. Nguyen et al. investigated the incidence of aspiration pneumonia during CRT for HNC; however, no data are available on this association [14]. We investigated the predictive factors of therapeutic response in HNC patients undergoing CRT or BRT and identified aspiration pneumonia as an independent predictive factor for complete response (CR). This result suggests that aspiration pneumonia during CRT or BRT has a detrimental effect on the treatment response. Furthermore, aspiration pneumonia leads to a low indication rate for R0 salvage surgery for patients with no CR or with recurrence after CRT or BRT. Aspiration pneumonia during CRT or BRT may cause treatment interruption or failure and subsequently prolong the overall treatment time; this in turn may result in reduced locoregional control [33]. Multivariate analysis in our study revealed a significant association between the occurrence of aspiration pneumonia and the risk of death. This is consistent with previous studies in which aspiration pneumonia was found to be a significant prognostic factor in patients with HNC [11, 34].

Our study has several limitations. First, although more than 350 people were subject to this analysis, this study is a retrospective study at a single institution. Second, it is sometimes difficult to differentiate aspiration pneumonia from other types of pneumonia. However, the diagnostic criteria for aspiration pneumonia used in this study are commonly used and are consistent with those prescribed by the Japanese Respiratory Society [35].

## Conclusions

We investigated the incidence of aspiration pneumonia during CRT or BRT in patients with locally advanced HNC. Four risk factors for aspiration pneumonia were identified: advanced N-classification, poor oral hygiene, hypoalbuminemia before treatment, and inpatient treatment. Aspiration pneumonia during CRT or BRT has a detrimental effect on treatment outcomes. Further prospective studies are required to validate the prognostic value of these risk factors in HNC patients receiving definitive CRT or BRT.

## Data Availability

The data that support the findings of this study are available from Shizuoka Cancer Center but restrictions apply to the availability of these data, which were used under license for the current study, and so are not publicly available. Data are however available from the authors upon reasonable request and with permission of Shizuoka Cancer Center.

## Abbreviations

3D-CRT: Conventional three-dimensional conformal radiation therapy
ALB: Serum albumin
BMI: Body mass index
BRT: Bio-radiotherapy
CR: Complete response
CRT: Chemoradiotherapy
Hb: Hemoglobin
HNCs: Head and neck cancers
HR: Hazard ratio
IMRT: Intensity-modulated radiation therapy
OR: Odds ratio
OS: Overall survival
RT: Radiotherapy
